# The novel cannabinoid receptor GPR55 mediates anxiolytic-like effects in the medial orbital cortex of mice with acute stress

**DOI:** 10.1186/s13041-017-0318-7

**Published:** 2017-08-11

**Authors:** Qi-xin Shi, Liu-kun Yang, Wen-long Shi, Lu Wang, Shi-meng Zhou, Shao-yu Guan, Ming-gao Zhao, Qi Yang

**Affiliations:** 1 Department of Pharmacology, School of Pharmacy, Fourth Military Medical University, Xi’an, China; 2 Department of Pharmacy, The 155th Central Hospital of PLA, Kaifeng, China; 3 Department of Neurobiology and Collaborative Innovation Center for Brain Science, Fourth Military Medical University, Xi’an, China

**Keywords:** GPR55, O-1602, Medial orbital cortex, Stress, Anxiety

## Abstract

The G protein-coupled receptor 55 (GPR55) is a novel cannabinoid receptor, whose exact role in anxiety remains unknown. The present study was conducted to explore the possible mechanisms by which GPR55 regulates anxiety and to evaluate the effectiveness of O-1602 in the treatment of anxiety-like symptoms. Mice were exposed to two types of acute stressors: restraint and forced swimming. Anxiety behavior was evaluated using the elevated plus maze and the open field test. We found that O-1602 alleviated anxiety-like behavior in acutely stressed mice. We used lentiviral shRNA to selectively knockdown GPR55 in the medial orbital cortex and found that knockdown of GPR55 abolished the anxiolytic effect of O-1602. We also used Y-27632, a specific inhibitor of ROCK, and U73122, an inhibitor of PLC, and found that both inhibitors attenuated the effectiveness of O-1602. Western blot analysis revealed that O-1602 downregulated the expression of GluA1 and GluN2A in mice. Taken together, these results suggest that GPR55 plays an important role in anxiety and O-1602 may have therapeutic potential in treating anxiety-like symptoms.

## Introduction

Depression and anxiety are the most prevalent neurological and psychiatric disorders affecting millions of people worldwide, with an estimated prevalence rate of 10–20% [1], and a tendency to increase. Stress is defined as any threat or perceived threat that disturbs an organism’s ability to maintain homeostasis. Although activation of stress response is initially adaptive, exposure to prolonged stress poses a significant risk for the development of numerous psychiatric disorders, including memory deficits [2], posttraumatic stress disorder [3, 4], and major depression [5, 6].

The endocannabinoid system is a neuromodulatory system that has been implicated in a wide range of physiological and pathological brain functions [7]. Clinical and animal studies consistently support the notion that the endocannabinoid system plays a central role in emotional homeostasis, stress responsiveness, energy balance, and cognitive function, whereas deregulation of the endocannabinoid signaling has been associated with neuropsychiatric conditions, such as depression, anxiety disorders, and schizophrenia [8, 9].

Recently, another G protein-coupled receptor, GPR55, was identified as a novel cannabinoid (CB) receptor owing to its high affinity for cannabinoid ligands, such as Δ^9^-tetrahydrocannabinol, 2-arachidonoylglycerol, anandamide, and rimonabant, independent of the CB_1_ and CB_2_ receptors (CB_1_R and CB_2_R) [10–13]. GPR55 was first identified in the human brain and liver [10]. The GPR55 gene has a widespread expression in the brain including the striatum, hippocampus, forebrain, cortex, and cerebellum [14]. Unlike the classical CB_1_R and CB_2_R signaling pathways, GPR55 is coupled to Gα_12/13_ [13, 15] and Gα_q_ proteins [16], and signals through ras homolog gene family member A (RhoA), Rho-associated protein kinase (ROCK), and phospholipase C (PLC) pathways. Increased intracellular Ca^2+^ triggers the activation of RhoA, Rac, and cdc42, which in turn induces the phosphorylation of extracellular-regulated protein kinase (ERK) [16–18]. Activation of GPR55 was reported to have regulatory roles in the central nervous system. For example, GPR55 regulates growth cone morphology and axon growth in the retina during development [19]. GPR55 knockout mice failed to develop mechanical hyperalgesia associated with inflammatory and neuropathic pain [20]. However, the exact role of GPR55 in the modulation of anxiety is unknown [21].

In the present study, the effects of GPR55 agonist and antagonist on stress-induced anxiety-like behaviors were evaluated. We first determined the expression level of GPR55 in emotion-related regions of the brain after chronic stress. Next, a panel of behavioral tests was used to examine the effect of GPR55 activation on anxiety-like symptoms. Lentiviral shRNA-mediated knockdown of GPR55 was used to confirm the effect of GPR55. Finally, we investigated the downstream pathway of GPR55 by using signal transduction antagonists. Our study results clarified the role of GPR55 in stress-induced mood disorders, and suggested that GPR55 may serve as a potential therapeutic target for the treatment of clinical anxiety or depression.

## Results

### Expression and distribution of GPR55 receptor in the cortex of chronic stress mice

**Fig. 1:**
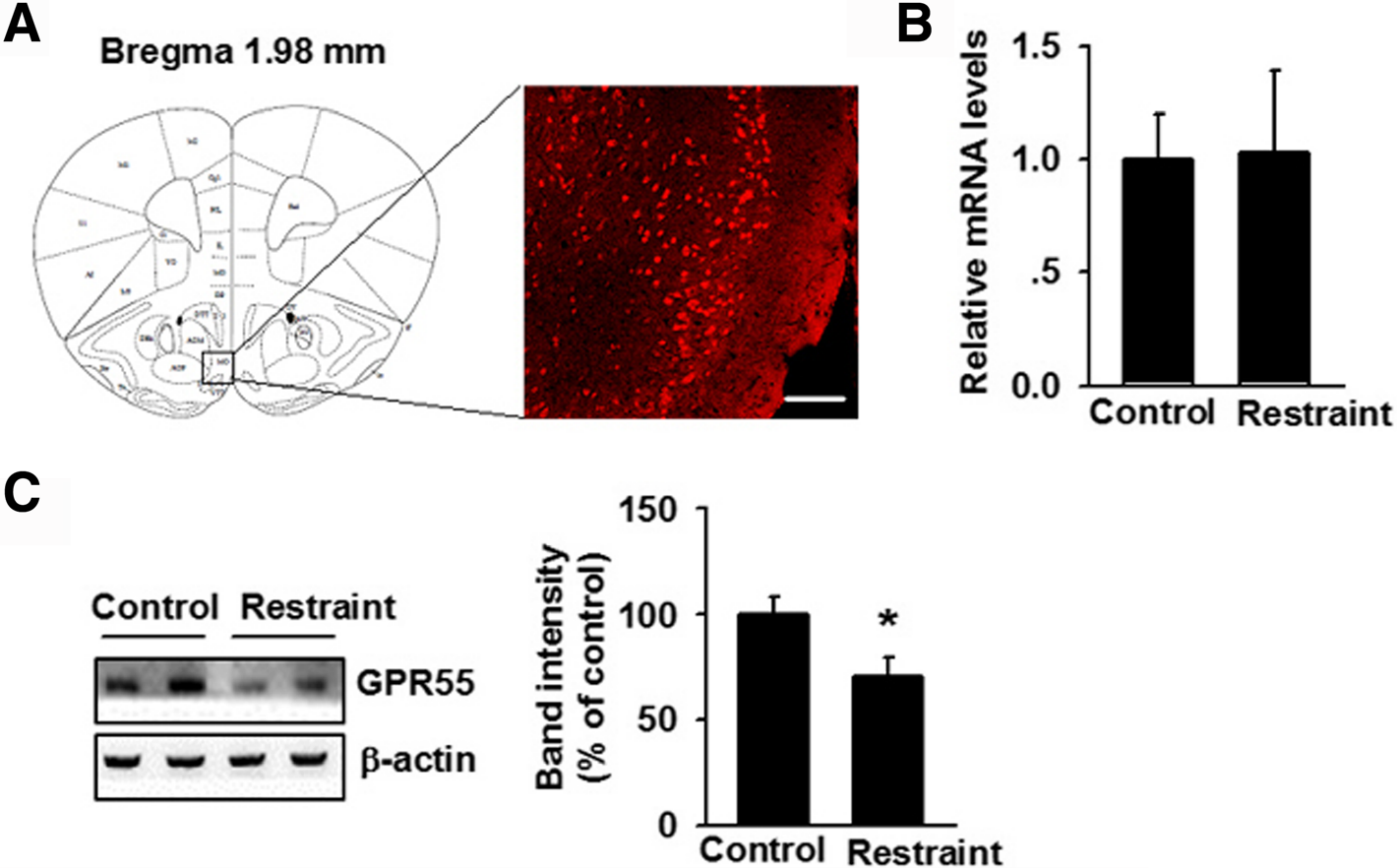
GPR55 expression in the medial orbital (MO) cortex. **a** Immunohistochemistry images showing GPR55 expression in the MO cortex. Scale bar = 100 μm. **b** The mRNA level of GPR55 in restraint-stressed mice did not change compared to that in the control group. **c** GPR55 expression decreased after daily exposure (4 h per exposure) to CRS for 21 consecutive days. *p < 0.05 versus control group. Each group contains 6–8 mice. Data are from three independent experiments

Chronic stress has been associated with impaired endocannabinoid system in the cortex of mice [22]. Through immunofluorescence staining, we observed that GPR55 was highly expressed in the MO cortex (Fig. 1a). Although CRS exposure did not alter the mRNA level of GPR55 (Fig. 1b), western blot analysis indicated that GPR55 expression significantly reduced after CRS exposure for 21 consecutive days (Fig. 1c). Downregulation of GPR55 expression in the MO cortex of CRS mice raises the possibility that GPR55 influences the development of anxiety, possibly acting as a compensatory response after stress.

### Effects of GPR55 agonist and antagonist in mice subjected to acute restraint stress

**Fig. 2:**
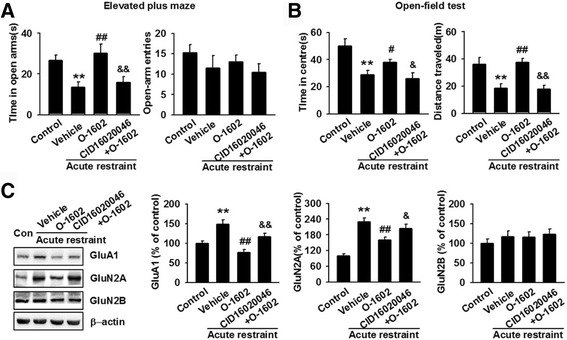
Intraperitoneal injection of O-1602 reverses acute restrain stress-induced anxiety-like behavior. **a** In the EPM, administration of O-1602 increased the time spent in the open arms compared to the vehicle group. O-1602 had no effect on the number of entries in the open arms. Treatment with CID16020046 and O-1602 decreased the time spent in the open arms and had no significant effect on the entries in the open arms compared to the O-1602 group. **p < 0.01 versus control group; ^##^ *p* < 0.01 versus vehicle group; ^&&^ *p* < 0.01 versus O-1602 group. **b** In the OFT, O-1602 treatment increased the time spent in the central area slightly and the total distance traveled significantly compared to the vehicle group. CID16020046 and O-1602 treatmentdecreased the total time spent in the central area and the total distance traveled as compared to the O-1602 group. *p < 0.05, **p < 0.01 versus control group; ^#^ *p* < 0.05, ^##^ *p* < 0.01 versus vehicle group; ^&^ *p* < 0.05, ^&&^ *p* < 0.01 versus O-1602 group. **c** Intraperitoneal injection of O-1602 reversed stress-induced expression of GluA1 and GluN2A, while GluN2B were unchanged. CID16020046 and O-1602 treatment abolished O-1602-mediated decrease in GluA1 and GluN2A expression. The expression of GluN2B were unchanged. **p < 0.01 versus control group; ^##^ *p* < 0.01 versus vehicle group; ^&^ *p* < 0.05, ^&&^ *p* < 0.01 versus O-1602 group. Each group contains 6–8 mice. Data are from three independent experiments

To investigate the influence and role of GPR55 in anxiety/depression-like behaviors, we used the acute restraint model. We intraperitoneally injected the GPR55 agonist O-1602 (10 mg/kg, 0.2 ml) into mice to induce anxiolytic effects. After 3 h, the mice were subjected to EPM and OFT. In the EPM test, acutely stressed mice treated with O-1602 spent more time in the open arms, although the number of entries into the open arms did not change significantly as compared to that reported for the vehicle group (Fig. 2a). To confirm the action of O-1602, the selective GPR55 antagonist CID16020046 (10 mg/kg, 0.2 ml) was simultaneously used with O-1602 to mice exposed to acute restraint stress. Compared to the O-1602-treated group, simultaneous injection of CID16020046 and O-1602 significantly decreased the duration in the open arms of the EPM, while the frequency in the open arms had no significant changes (Fig. 2a). Results from the OFT showed that the time of O-1602 treatment spent in the central area increased slightly, whereas the total distance traveled increased significantly as compared to the vehicle group (Fig. 2b). Meanwhile, compared to the O-1602-treated group, CID16020046 and O-1602 treatment slightly decreased the time spent in the central area of the OFT and significantly decreased the total distance traveled (Fig. 2b). In addition, we tested the expression levels of several glutamate receptors, because a lot of studies suggested the glutamate receptors have a strong association with anxiety. Western blot analysis showed that intraperitoneal injection of O-1602 prevented acute stress-induced increase in GluA1 and GluN2A expression, but not in GluN2B (Fig. 2c). Simultaneous injection administration of CID16020046 and O-1602 abolished O-1602-mediated decrease in GluA1 and GluN2A expression. The expressions of GluN2B were not changed in the MO cortex of stressed mice exposed to CID16020046 and O-1602 treatment (Fig. 2c). Overall, these results suggest that GPR55 play an important role in anxiety/depression-like behaviors and activation of GPR55 can reverse anxiety/depression-like behaviors after acute stress.

### Effects of GPR55 agonist and antagonist in mice exposed to forced swimming stress

**Fig. 3:**
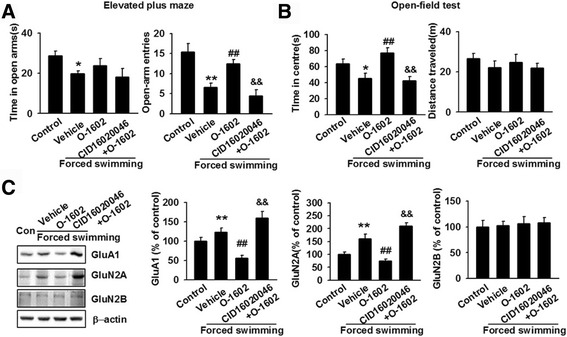
Intraperitoneal injection of O-1602 reverses forced swimming-induced anxiety-like behavior. **a** In the EPM, administration of O-1602 increased the number of entries in the open arms and the time into the open arms did not change significantly as compared to the vehicle group. CID16020046 and O-1602 decreased the frequency in the open arms and had no significant changes on the duration in the open arms as compared to the O-1602 group. *p < 0.05, **p < 0.01 versus control group; ^##^ *p* < 0.01 versus vehicle group; ^&&^ *p* < 0.01 versus O-1602 group. **b** In the OFT, administration of O-1602 significantly increased the time spent in the central area and had no effect on the total distance traveled. CID16020046 and O-1602 treatment decreased the time spent in the central area, while it had no significant effect on the total distance traveled. *p < 0.05 versus control group; ^##^ *p* < 0.01 vehicle group; ^&&^ *p* < 0.01 versus O-1602 group. **c** Administration of O-1602 reversed stress-induced expression of GluA1 and GluN2A, but not GluN2B. CID16020046 and O-1602 treatment abolished O-1602-mediated decrease in GluA1 and GluN2A expression, while had no effect on the expression of GluN2B. **p < 0.01 versus control group; ^##^ *p* < 0.01 versus vehicle group; && *p* < 0.01 versus O-1602 group. Each group contains 6–8 mice. Data are from three independent experiments

In order to confirm the importance of GPR55 in acute stress, the forced swimming model was used. In the EPM test, stressed mice injected with O-1602 increased the number of entries into the open arms significantly, although the time into the open arms did not change significantly as compared to the vehicle group (Fig. 3a). Meanwhile, simultaneous injection of CID16020046 and O-1602 significantly decreased the frequency in the open arms of the EPM as compared to that reported for the O-1602 group, while the duration in the open arms had no significant changes (Fig. 3a). In addition, compared to the vehicle group, the O-1602 group increased the time spent in the central area significantly in the OFT, whereas the total distance traveled did not change (Fig. 3b). CID16020046 and O-1602 group significantly decreased the time spent in the central area of the OFT as compared to the O-1602 group and had no effect on the total distance traveled (Fig. 3b). Western blot analysis showed O-1602 treatment prevented stress-induced increase in GluA1 and GluN2A expression in the MO cortex of mice, while the expression of GluN2B was unchanged (Fig. 3c). Simultaneous injection of CID16020046 and O-1602 abolished O-1602-mediated decrease in GluA1 and GluN2A expression in the MO cortex, while the expression of GluN2B had no significant changes (Fig. 3c). Overall, these results further confirm that GPR55 is involved in anxiolytic response and that pharmacological enhancement of GPR55 function can reverse anxiety/depression-like behaviors after acute stress.

### Role of GPR55 activation in O-1602-mediated anxiolytic effects

**Fig. 4:**
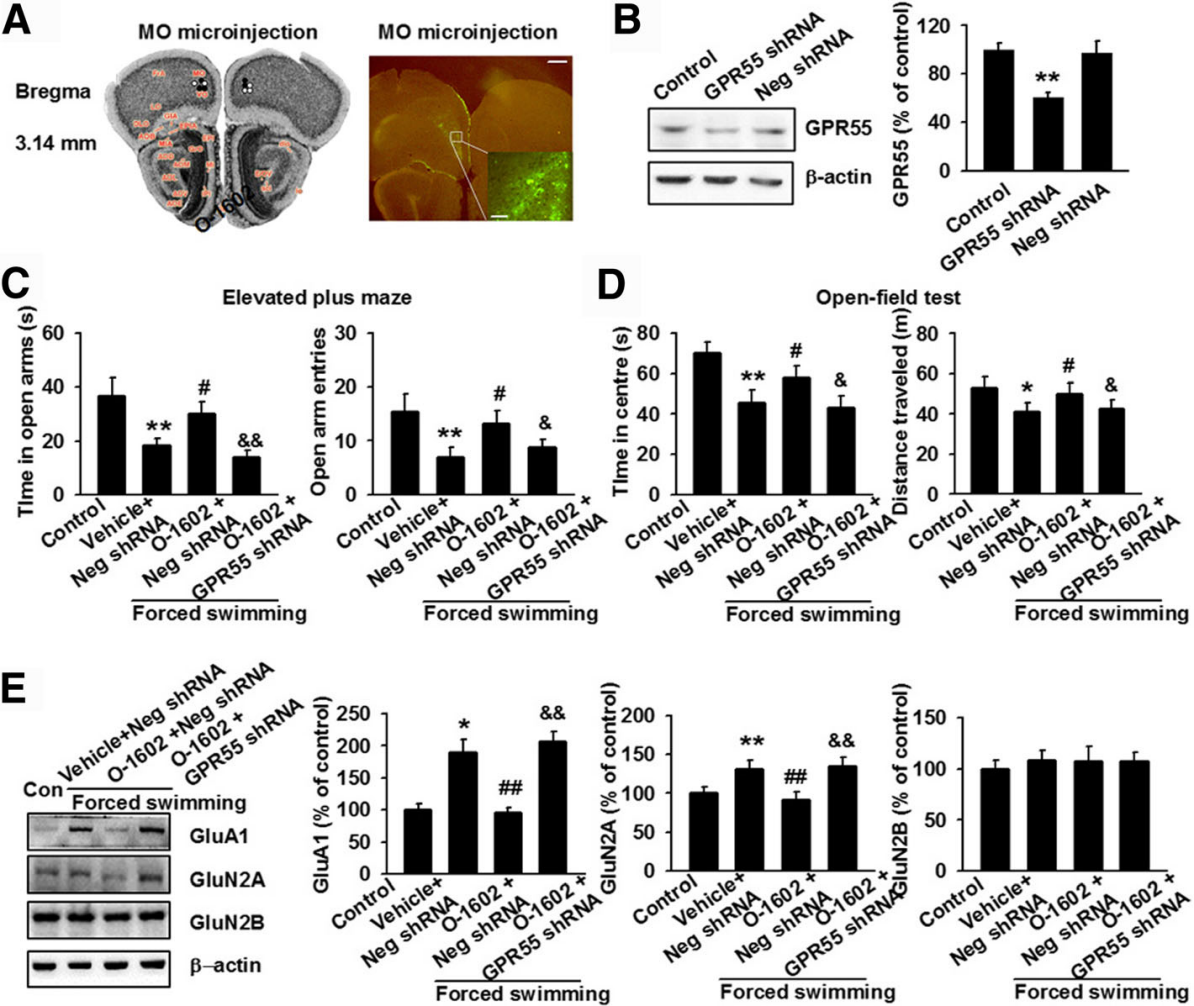
GPR55 knockdown abolished O-1602-mediated anxiolytic effects. **a** Immunohistochemistry image showing the GPR55 shRNA infected cells (GFP positive) in the MO cortex (Scale bar, 500 um; insert: scale bar, 100 um). **b** Western blot image showing the expression of GPR55 in GPR55 knockdown (GPR55 shRNA-injected group) in the MO cortex as compared to negative shRNA-injected group. **p < 0.01 versus control group; **c** In the EPM, O-1602 with negtive shRNA increased the duration and frequency in the open arms. O-1602 with GPR55 shRNA abolished the increase on duration and frequency in the open arms. **p < 0.01 versus control group; ^#^ *p* < 0.05 versus vehicle + negative shRNA group; ^&^ *p* < 0.05, ^&&^ *p* < 0.01 versus O-1602 + negative shRNA group. **d** In the OFT, O-1602 with negtive shRNA increased the time in the center area and the total distance traveled. O-1602 with GPR55 shRNA reversed the effect on time in the center area and the total distance traveled. *p < 0.05, **p < 0.01 versus control group; ^#^ *p* < 0.05 versus vehicle + negative shRNA group; ^&^ *p* < 0.05versus O-1602 + negative shRNA group. **e** O-1602 with negtive shRNA decreased the expression of GluA1 and GluN2A, but GluN2B did not decrease. O-1602 with GPR55 shRNA abolished the decrease in GluA1 and GluN2A expression, although knockdown did not affect the expression of GluN2B. *p < 0.05, **p < 0.01 versus control group; ^##^ *p* < 0.01 versus vehicle group; ^&&^ *p* < 0.01 versus O-1602 + negative shRNA group. Each group contains 6–8 mice. Data are from three independent experiments

Previous studies have associated CB_1_R and CB_2_R with anxiety/depression-like behaviors [23, 24]. In order to exclude the possible involvement of CB_1_R and CB_2_R in O-1602-mediated anxiolytic effect, a lentiviral shRNA specific for GPR55 was constructed and stereotaxically microinjected into the MO cortex of mice at a concentration of 10^9^ TU/ml. After 7 days of infection, cells were labeled green by GFP (Fig. 4a). Western blot analysis confirmed the efficiency of knockdown, which resulted in a 62.7 ± 4.1% reduction in the GPR55 protein band intensity (Fig. 4b). In the EPM, O-1602 with negtive shRNA increased the time in the open arms and the number of entries into the open arms significantly, but the increase was reversed by O-1602 with GPR55 shRNA (Fig. 4c). Results from the OFT showed that the O-1602 with negtive shRNA increased the time spent in the central area and the total distance traveled. However, O-1602 with GPR55 shRNA prevented the behavioral improvement mediated by O-1602 with negtive shRNA (Fig. 4d). Moreover, O-1602 with negtive shRNA decreased the expression of GluA1 and GluN2A, but GluN2B did not decrease. O-1602 with GPR55 shRNA abolished the decrease in GluA1 and GluN2A expression, although no change in the expression of GluN2B was observed (Fig. 4e). These observations show that GPR55 plays an important role in the development of anxiety/depression-like behaviors.

### Possible signaling pathways involved in O-1602-induced anxiolytic effect

**Fig. 5:**
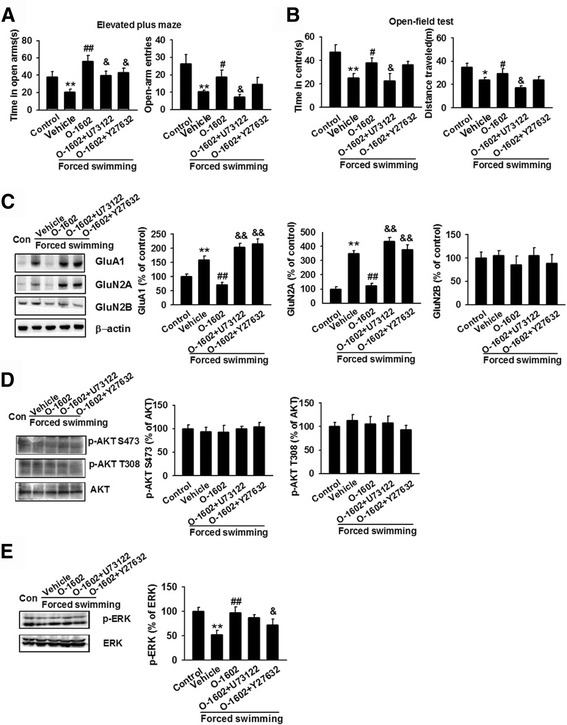
Administration of Y-27632 and U73122 abolished the effect of O-1602. **a** In the EPM test, co-administration of Y-27632 or U73122 with O-1602 decreased the time spent and the number of entries in the open arms. **p < 0.01 versus control group; ^#^ *p* < 0.05, ^##^ *p* < 0.01 versus vehicle group; ^&^ *p* < 0.05 versus O-1602-treated group. **b** In the OFT, co-administration of Y-27632 or U73122 with O-1602 decreased the time in the central area and the total distance traveled. *p < 0.05, **p < 0.01 versus control group; ^#^ *p* < 0.05 versus vehicle group; ^&^ *p* < 0.05 versus O-1602-treated group. **c** Administration of Y-27632 or U73122 reversed O-1602-induced expression of GluA1, GluN2A, and GluN2B. *p < 0.05, **p < 0.01 versus control group; ^##^ *p* < 0.01 versus vehicle group; ^&^ *p* < 0.05, ^&&^ *p* < 0.01 versus O-1602 group. **d** Administration of Y-27632 or U73122 had no effect on the phosphorylation of AKT at S473 and T308. **e** Administration of Y-27632 and U73122 decreased O-1602-induced expression of p-ERK. **p < 0.01 versus control group; ^##^ *p* < 0.01 versus vehicle group; ^&^ *p* < 0.05 versus O-1602 group. Each group contains 6–8 mice. Data are from three independent experiments

The downstream signaling cascade by which GPR55 agonist initiates its effect was investigated using selective PLC inhibitor U73122 and RhoA/ROCK inhibitor Y-27632. Simultaneous injection of U73122 (10 mg/kg, 0.2 ml) and O-1602 significantly decreased the duration and frequency in the open arms of the EPM as compared to O-1602 treatment alone (Fig. 5a). In the OFT, the time spent in the central area and the total distance traveled also significantly decreased (Fig. 5b). Meanwhile, simultaneous injection of Y-27632 (30 mg/kg, 0.15 ml) and O-1602 slightly decreased the duration and frequency in the open arms of the EPM, as well as the time spent in the central area and the total distance traveled in the OFT (Fig. 5a and b). Overall, although both Y-27632 and U73122 reversed the anxiolytic effects of O-1602, the reversal effect of U73122 is more effective than that of Y-27632. Immunoblot analysis showed that both Y-27632 and U73122 abolished O-1602-mediated decrease in GluA1 and GluN2A expression in the MO cortex, while the expression of GluN2B increased slightly but did not reach statistical significance (Fig. 5c). Next, we investigated the effects of Y-27632 and U73122 on AKT and ERK phosphorylation. Both inhibitors reduced p-ERK (Fig. 5e) but had no effect on total AKT and AKT phosphorylation at both Thr308 and Ser473 (Fig. 5d). These results show that both PLC-PKC and RhoA-ROCK pathways are involved in GPR55 activation, leading to ERK phosphorylation.

## Discussion

The orphan G-protein coupled receptor, GPR55, is described as an atypical cannabinoid receptor that can be activated by lysophosphatidylinositols and certain synthetic or endogenous cannabinoid molecules [13]. Therefore, the GPR55 receptor plays an important role in the pharmacological actions of cannabinoids. However, the exact role of the GPR55 receptor in the central nervous system, especially in anxiety, warrants further investigation. In the present study, we focused on the role of GPR55 activation in modulating anxiolytic-like effects.

Previous studies have shown that GPR55 mRNA/protein is expressed in several brain areas such as the hippocampus, hypothalamus, frontal cortex, and cerebellum [25]. We found that GPR55 was expressed in the MO cortex, which is considered an important region that controls mood and cognitive functions [26]. MO cortex is strongly connected to the hippocampus and associated areas of the cingulate, retrosplenial, and entorhinal cortices, anterior thalamus, and septal diagonal band [27]. It has been suggested that the MO cortex is involved in the process of decision-making. Activity in the MO cortex was also detected when suppressing negative emotions, especially in approach-avoidance situations [28]. Chronic stress is a risk factor for the development of mood disorders [29], and can also disrupt the MEK/ERK signaling in the MO cortex [30]. In this study, we used a 21-day restraining protocol to induce chronic stress. With this protocol, we observed a significant decrease in the protein expression of GPR55 in the MO cortex, although GPR55 mRNA level remained unchanged. To our knowledge, this is the first study reporting the role of GPR55 in the development of anxiety/depression.

In a previous study in which the GPR55 agonist O-1602 was used, it was shown that activation of GPR55 relieved anxiety-like behaviors in normal rats [21]. However, it is unknown whether similar anxiolytic effects can be observed in stress-induced mice and the role GPR55 plays under these pathological conditions. Therefore, we used two kinds of acute stress models, restraint and forced swimming, to induce anxiety-like behaviors, which were measured via EPM and OFT. Restraint has been widely characterized as an acute stressor as it is a simple experimental procedure with high reproducibility [31, 32], while forced swimming was adopted as a stressor because its neurochemical and hormonal aspects satisfy the stress criteria in this study [33, 34]. In our study, both restraint and forced swimming decreased the time spent in the open arms and central area, and increased GluA1 expression in the MO cortex of mice. These changes were reversed by the GPR55 agonist O-1602, and the GPR55 antagonist CID16020046 were able to abolish O-1602-mediated anxiolytic effect. Our results are consistent with the known anxiolytic effects of O-1602 mediated through GPR55 activation, as determined previously by behavioral tests [21].

We also investigated the involvement of glutamate receptors during stress-induced anxiety. A large number of clinical and preclinical studies have demonstrated the important role of glutamate in the pathophysiology of anxiety disorders [35–38]. Likewise, findings from animal studies have established a strong association between anxiety and glutamate receptors [39–41]. Ionotropic glutamate receptors include the α-amino-3-hydroxy-5-methyl-4-isoxazolepropionic acid (AMPA) and *N*-Methyl-D-aspartic acid (NMDA) receptors, such as GluA1, GluA2, GluN2A, and GluN2B, which play crucial roles in regulating synaptic neurotransmission and plasticity [42]. Our study indicated that acute stress results in increased baseline expression of GluA1 and GluN2A in the MO cortex, with no alterations in GluN2B expression. A related study also showed negative correlation between the time spent in the open arms of the EPM and the protein levels of glutamate receptors [43]. In addition, several studies have shown that the AMPA and NMDA receptor antagonists are effective anxiolytics over a wide range of animal models of anxiety [44–46]. These results may indirectly support our findings that anxiety-like behavior is related to reduced GluA1 and GluN2A levels in the MO cortex. In addition, we demonstrated that stress-induced suppression of glutamate receptor expression could be reversed by O-1602 treatment. To date, there are no reports implicating the role of glutamate receptors in GPR55 activity, and a previous study only demonstrated that GPR55 co-localized with the synaptic vesicle protein vesicular glutamate transporter 1 in the stratum radiatum [14]. Thus, we are the first group to establish the relationship between glutamate receptors and GPR55-mediated anxiolytic effects.

In accordance with the diverse and complex pharmacology of GPR55, the current literature regarding the downstream signaling of the receptor is equally disparate. There is growing evidence that GPR55 couples to Gα_13_, Gα_12_, or Gα_q_ in the GTPγS assay [13, 15, 16], but not to Gα_i/o_ protein, which is coupled to CB_1_R and CB_2_R [47, 48]. We hypothesized that GPR55 activation triggers the activation two separate downstream signaling cascades, namely the Gα_12/13_-RhoA-ROCK and Gα_q_-PLC-PKC pathways, both of which have been shown to play important physiological roles in other G protein-coupled receptors [49, 50]. Our results indicated that some anxiolytic effects induced by O-1602 in the acute stress model can be attenuated by Y-27632, a specific inhibitor of ROCK, and U73122, an inhibitor of PLC. Thus, activation of GPR55 links the RhoA-ROCK and PLC-PKC pathways to the development of mood disorders, evident by alteration of the expression of glutamate receptors.

Although involvement of the mitogen-activated protein kinase (MAPK) signaling, elevation of calcium levels, and expression of transcription factors initiated by GPR55 activation have been reported in various papers, the converging pathway is the activation of MAPK, which results in the phosphorylation of ERK [51]. ERK plays a crucial role in regulating mood-related phenotypes and participates in the antidepressant response in various brain regions [52–54]. Mice exposed to CRS exhibited depressive-like behavior along with reduced MAPK/ERK signaling in the MO cortex and dorsal endopiriform nuclei of the prefrontal cortex [30]. Although altered ERK signaling in the cortex of mice with anxiety/depression has been documented, it is unclear whether GPR55 plays any modulatory role. In the current study, phosphorylation of ERK markedly decreased in the MO cortex of acute stress-induced mouse brains, and this reduction was blocked in O-1602-treated mice, which corresponded with the lack of anxiety-like behavioral responses. In contrast, phosphorylation of AKT was not altered. All of these observations suggest that GPR55 activation induces ERK signaling which mediates O-1602-induced anxiolytic effects. However, we cannot rule out the possible involvement of other signaling pathways, such as p38, NFAT, and Rac [15, 17, 18].

Interestingly, GPR55 knockout mice were reported to have similar anxiety-like behaviors as wild-type mice [55]. This inconsistent result may be due to the compensatory increase in homologous superfamily after genetic deletion. Nevertheless, results obtained for the O-1602-treated mice we used are consistent with its known activity on GPR55 [21, 56]. However, recent studies have suggested targets other than GPR55 for O-1602. For example, it was reported that O-1602 also has affinity for GPR18 receptors [56]. Although other studies have attempted to determine the specificity of O-1602 for GPR55 by using the GPR55 antagonist ML193 [21, 57], the question regarding its specificity still remains. Because ML193 may also antagonize CB_1_R or GPR35 at higher doses [58], we used lentiviral shRNA to selectively knockdown GPR55 in the MO cortex by stereotactic microinjection. We found that GPR55 knockdown abolished the anxiolytic effect of O-1602. Therefore, our work confirms the specific role of GPR55 in the modulation of anxiety.

Taken together, the present findings show that O-1602 ameliorated anxiety-like symptoms and reversed stress-induced suppression of glutamate receptor expression through GPR55 activation. These data support the notion that GPR55 is a neurobiological target in anxiety- and stress-related disorders. Future studies may reveal whether GPR55 shows anxiolytic effect in other models of stress-induced anxiety, such as predator scent stress or chronic unpredictable stress.

## Methods

### Animals

Adult male C57BL mice (6–8 weeks of age) were used in all experiments, and were obtained from the Laboratory Animal Center of the Fourth Military Medical University (FMMU). The animals were housed in plastic boxes in groups of six with food and water available ad libitum in a colony room with controlled temperature (24 ± 2 °C), humidity (50–60%), and a light cycle from 8:00 A.M. to 8:00 P.M. under laminar airflow. The mice were given commercial chow diets and allowed to adapt to laboratory conditions for at least 1 week before the start of experiments. All animal protocols were approved by the Fourth Military Medical University Animal Care and Use Committee.

### Drug

All drugs used in this study were purchased from Tocris Bioscience (Missouri, USA). The GPR55 agonist O-1602 and the selective GPR55 antagonist CID16020046 were dissolved in 10% dimethyl sulfoxide (DMSO) at a concentration of 1 mg/ml. Y-27632 was dissolved in distilled water at a concentration of 4 mg/ml. U73122 was dissolved in a mixture of Tween-20: DMSO: normal saline (1: 49: 50 ratio) at a concentration of 1 mg/ml. All drugs were stored at −20 °C. The drugs were given immediately after acute stress in mice. We intraperitoneally injected the O-1602, CID16020046 and Y-27632 into mice. The U73122 was given by intragastric administration.

### Immunohistochemistry

Mice were anesthetized with pentobarbital sodium and perfused with sterile saline, followed by 4% paraformaldehyde. The brain was post-fixed in 4% paraformaldehyde for 6 h at 4 °C, and then transferred to 20% sucrose for 48 h. A coronal section including the medial orbital (MO) cortex was cut using a microtome-cryostat (Leica, Heidelberg, Germany) and processed for immunostaining. After blocking with normal goat serum containing 0.1% Triton X-100 for 30 min, sections were incubated overnight with rabbit anti-GPR55 (1:200; Abcam, Cambridge, MA; ab203663) primary antibody at 4 °C. Subsequently, the sections were rinsed with PBS three times and then incubated with Cy3-conjugated anti-rabbit secondary antibody (1:200; Boster Bio-Technology, Wuhan, China). Sections were visualized using a FV1000 confocal laser microscope (Olympus, Tokyo, Japan).

### Chronic restraint stress (CRS)

Mice were restrained with restrainers constructed of clear plastic tubes (height: 5 cm, width: 5.5 cm, length: 22 cm) without physical compression or pain, 4 h daily for 21 consecutive days [59]. Mice were deprived of food and water during restraint.

### Acute stress

Restraint *(Model 1)* and forced swimming *(Model 2)* are two types of stressors used extensively to induce anxiety [60, 61]. In the acute stress model, mice were subjected to either restraint or forced swimming. After acute stress, the mice were placed in plastic boxes with food and water available ad libitum without restraint. Mice were housed in the same experimental room during the stress period. After 24 h, the mice were subjected to two behavioral tests: open field test (OFT) and elevated plus maze (EPM).

### Restraint (model 1)

In the restraint model, mice were restrained with restrainers constructed of clear plastic tubes (height: 5 cm, width: 5.5 cm, length: 22 cm) without physical compression or pain, 4 h daily for 2 consecutive days.

### Forced swimming (model 2)

In the forced swimming experiment, mice were individually placed in an open cylindrical container (diameter: 10 cm, height: 25 cm) containing 20 cm of water at 20 ± 1 °C for 15 min. This depth forced the mice to swim without allowing their tails to touch the bottom of the container. Mice were forced to swim 15 min daily for 2 consecutive days. At the end of each session, the mice were removed from the water, and immediately and gently wiped dry.

### Elevated plus maze (EPM)

The apparatus was made of grey plastic and consisted of two opposing open arms (25 × 8 × 0.5 cm) and two closed arms (25 × 8 × 12 cm) that extended from a common central platform (8 × 8 cm). The apparatus was elevated to a height of 50 cm above the floor. Mice were allowed to habituate in the testing room for 2 days before the test, and were pretreated with gentle handling twice per day to minimize nervousness. Mice were adapted to apparatus for the 3 min before the experiment. For each test, individual animals were placed in the center square, facing an open arm, and allowed to move freely for 5 min. Mice were videotaped using a camera fixed above the maze and analyzed using a video tracking system. Open and closed arm entries (all four paws in an arm) were scored by an experienced observer. The number of entries and time spent in each arm were recorded. After each test, the EPM was carefully cleaned with 75% ethanol and allowed to dry.

### Open-field test (OFT)

The open field consisted of a square arena (30 × 30 × 30 cm^3^) with clear Plexiglas walls and floor placed inside an isolation chamber with dim illumination and a fan. Mice were placed in the center of the box and allowed to adjust to the environment for 10 min. Mice were videotaped using a camera fixed above the floor and analyzed with a video tracking system. The “center” field is defined as the central area (15 × 15 cm^2^) of the open field, one-fourth of the total area. Each subject was placed in the center of the open field, and its activity was measured for 5 min.

### Western blot analysis

After behavioral testing, all mice were anesthetized with an overdose of pentobarbital sodium, and then decapitated. The MO cortex tissue was chopped into small pieces and homogenized in ice-cold RIPA lysis buffer containing 1× protease inhibitor cocktail. Equal amounts of protein were resolved using 9% sodium dodecyl sulfate-polyacrylamide electrophoresis (SDS-PAGE) gel and transferred to a nitrocellulose membrane. The membrane was then incubated with primary antibodies overnight at 4 °C. The following antibodies were used: anti-GPR55 (1:200; Abcam, ab203663), anti-GluA1 (1:1000; Abcam, ab31232), anti-GluN2A (1:1000; Abcam, ab133265), anti-GluN2B (1:400; Millipore, Billerica, MA; MAB5780), anti-β-actin (1:10,000; Sigma, St Louis, MO; A5316), anti-ERK (1:1000; ZSGB-BIO, Beijing, China; L2115), anti-p-ERK (1:1000; ZSGB-BIO, J2114), anti-AKT (1:1000; Cell Signaling, Danvers, MA; 4691), anti-p-AKT (Thr308) (1:1000; Cell Signaling, 13,038), and anti-p-AKT (Ser473) (1:1000; Cell Signaling, 9271). The membranes were incubated with horseradish peroxidase-conjugated secondary antibodies (anti-rabbit/anti-mouse IgG for the primary antibodies), and bands were visualized using enhanced chemiluminescence (ECL, GE Healthcare Pharmacia). Densitometric analysis of Western blots was conducted using a ChemiDoc XRS (Bio-Rad, Hercules, CA, USA) and quantified using Quantity One version 4.1.0 (Bio-Rad). Band intensity of target proteins was expressed as percentage relative to the control.

### RNA preparation and RT-qPCR

Total RNA was extracted from cultured neurons and prefrontal cortex using the RNeasy mini kit (Qiagen, Valencia, CA). Reverse transcription-polymerase chain reaction (RT-PCR) was performed on 1 μg of RNA using the PrimeScript RT reagent kit with gDNA Eraser (TaKaRa Biotechnology, Dalian, China) to generate cDNA. Following synthesis, the cDNA and primers were mixed with 2× SYBR Premix Ex TaqII (TaKaRa Biotechnology, Dalian, China), and quantitative real-time PCR was performed using the ABI PRISM 7500 Sequence Detection System (Applied Biosystems, Warrington, UK). The following primer sequences were used: 5′-AGGCTATCTTCACCAAGCAGCAC-3′ (forward) and 5′-TGGTTCAGCTGTCTGCCATTTC-3′ (reverse) for *gpr55*, and 5′-TGTGTCCGTCGTGGATCTGA-3′ (forward) and 5′- TTGCTGTTGAAGTCGCAGGAG-3′ (reverse) for *gapdh*, which served as the internal control. The relative amounts of mRNA were calculated using the comparative threshold cycle method. The thermal cycling conditions were as follows: 95 °C for 30 s, followed by 40 cycles of 95 °C for 5 s, and 60 °C for 34 s.

### Intracerebral shRNA lentivirus infusion

Mice were anesthetized using intraperitoneal injection of pentobarbital sodium (30 mg/kg). The GPR55 shRNA lentivirus (10^9^ TU/ml) was stereotaxically microinjected into the MO (3.14 mm anterior to bregma, ± 0.1 mm lateral to midline, and 2.5 mm ventral to bregma) at a rate of 0.2 μl/min for 5 min, resulting in a dose of 1 μl of lentivirus. GPR55 lentiviral vectors with a green fluorescent protein (GFP) tag were constructed by Genepharma (Shanghai, China). To generate the GPR55 shRNA, a target sequence was designed against mouse GPR55: 5′-AGATCTTTGGCTTCCTCCTTCCCAT-3′. After microinjection, the hole was sealed with bone wax, and the wound was sutured. The mice were used for subsequent experiments 1 week after surgery.

### Statistical analysis

The data were expressed as mean ± standard error of the mean (SEM). Statistical comparisons were performed via analysis of variance (ANOVA). If the ANOVA was significant, post hoc comparisons were conducted using Tukey’s test. In all cases, *p* < 0.05 was considered statistically significant.

## Abbreviations

AMPA: α-amino-3-hydroxy-5-methyl-4-isoxazolepropionic acid
ANOVA: Analysis of variance
CB: Cannabinoid
CRS: Chronic restraint stress
DMSO: Dimethyl sulfoxide
ECL: Enhanced chemiluminescence
EPM: Elevated plus maze
ERK: Extracellular-regulated protein kinase
GFP: Green fluorescent protein
GPR55: G protein-coupled receptor 55
MAPK: Mitogen-activated protein kinase1
MO: Medial orbital
NMDA: N-Methyl-D-aspartic acid
OFT: Open field test
PLC: Phospholipase C
RhoA: Ras homolog gene family member A
ROCK: Rho-associated protein kinase
RT-PCR: Reverse transcription-polymerase chain reaction
SDS-PAGE: Sodium dodecyl sulfate-polyacrylamide electrophoresis
SEM: Standard error of the mean
